# Retracted: Generating Personalized Web Search Using Semantic Context

**DOI:** 10.1155/2017/1295378

**Published:** 2017-07-16

**Authors:** The Scientific World Journal

The Scientific World Journal has retracted the article titled “Generating Personalized Web Search Using Semantic Context” [1]. The peer review of the article has been compromised, due to a conflict of interest between the handling editor and one of the authors.
